# The pituitary adenylate cyclase‐activating polypeptide receptor and its splice variants: Unique roles in affective and motivated behavior

**DOI:** 10.1111/jne.70186

**Published:** 2026-04-14

**Authors:** Brody A. Carpenter, Jessica R. Barson

**Affiliations:** ^1^ Department of Neurobiology and Anatomy Drexel University College of Medicine Philadelphia Pennsylvania USA

**Keywords:** alcohol, hop, null, short, stress

## Abstract

Pituitary adenylate cyclase‐activating polypeptide (PACAP) is a pleiotropic neuropeptide with established roles in stress, affective behavior, and motivated behavior. Its primary receptor in the brain, the PACAP type I receptor (PAC1), has multiple variants due to alternative splicing of the gene, and these variants have been found to have different relationships with the stress response. In the field of motivated behavior, however, there has been much more limited research on these variants. This review focuses on the PAC1 and its splice variants, to propose that they should be thoroughly characterized in the context of motivated behavior. It develops the hypothesis that, for the motivated behavior of drug use, upregulation of a specific receptor variant during repeated episodes of drug use and withdrawal serves to reverse the early relationship between PACAP and drug use, switching from negative feedback in a non‐dependent state to positive feedback in a dependent state. The review will first examine the known brain distribution and receptor dynamics of the PAC1 variants. Next, it will examine the known roles of PACAP and its receptor variants in stress, anxiety, and depression. Then, it will describe the known role of the PACAP system in alcohol use, as an example of drug use. Finally, the review will consider these known relationships in order to advance the proposal about how the PAC1 receptor variants may interact with drug use and dependence. Further research on this relationship could allow for the development of novel and effective medications for the treatment of drug use disorders.

## 1 INTRODUCTION

Pituitary adenylate cyclase‐activating polypeptide (PACAP) is a pleiotropic neuropeptide that has received significant attention across fields ranging from feeding to traumatic brain injury to immune function.^1^, ^2^, ^3^, ^4^ Discovered in 1989 as a potent activator of adenylate cyclase, which increased accumulation of cyclic adenosine monophosphate (cAMP),^5^ PACAP is now known to act not only as a hormone, but also as a neurohormone, neurotransmitter, and trophic factor,^3^ and to participate in functions such as brain development, neuroprotection, and regulation of the adrenal gland, among others.^6^ While PACAP may be one of the most studied neuropeptides, its primary receptor in the brain, the PACAP type I receptor (PAC1), has received much more limited attention, at least in the field of motivated behavior. Complicating a full understanding of PAC1, the receptor has been found to have multiple variants due to alternative splicing of the gene (*ADCYAP1R1* or *Adcyap1r1*), and different publications over the years have used different terminology to refer to these splice variants.

In this review, we focus on the PAC1 and its splice variants to examine their known distribution and actions in the brain and known function in the stress response and affective behavior, in order to propose that the PAC1 splice variants should also be thoroughly examined in the context of motivated behavior. In light of the growing literature on the role of PACAP in alcohol use, we provide an in‐depth review of the known involvement of the PACAP system in this specific motivated behavior, and we postulate about a possible relationship between the PAC1 splice variants and drug use and dependence.

## 2 EXPRESSION AND DISTRIBUTION OF PACAP

The neuropeptide, PACAP, is part of the glucagon/secretin/vasoactive intestinal polypeptide (VIP) superfamily of neuropeptides.^5^ As with other neuropeptides, it is found in large, dense‐core vesicles, and thought to be released from neurons upon high‐frequency or burst stimulation.^7^ Highly conserved across species, the PACAP precursor (prepro‐PACAP) consists of 173–176 amino acid residues, having been characterized in animals ranging from tunicates and frogs, to mice and rats, to marmosets and humans.^2^, ^6^, ^8^ While PACAP is highly expressed in the brain, it should be noted that it is also expressed in a number of peripheral organs.^3^ In the brain, it is primarily expressed in neurons, but it has also been found in glial cells.^9^

In rodents, cell bodies containing PACAP transcript and peptide (using in situ hybridization and immunohistochemistry) have been identified in a multitude of brain regions, many of which are part of the limbic system. These include regions such as nuclei of the hypothalamus (e.g., paraventricular nucleus, supraoptic nucleus, medial preoptic area, suprachiasmatic nucleus, arcuate nucleus, dorsomedial nucleus, ventromedial nucleus, lateral hypothalamic area), thalamus (e.g., paraventricular nucleus, nucleus reuniens, mediodorsal nucleus), prefrontal cortex (e.g., anterior cingulate, prelimbic, infralimbic), amygdala (e.g., basolateral nucleus, basomedial nucleus), bed nucleus of the stria terminalis, habenula, ventral tegmental area, locus coerulus, and periaqueductal gray.^4^, ^10^ It should be noted that the in situ hybridization method used to identify PACAP soma in the brain had located it in regions that were not previously detected with immunohistochemistry.^4^ Levels of PACAP have been found to exhibit sex‐related differences in the brain. While no differences in PACAP transcript or peptide have been observed in the rat hypothalamus,^11^, ^12^, ^13^ female mice and rats have been found to have higher PACAP transcript and peptide levels than males in both the paraventricular nucleus of the thalamus and the bed nucleus of the stria terminalis.^14^, ^15^ This distribution of PACAP, coupled with the sex‐related differences in PACAP levels in the brain, suggests that PACAP is positioned to mediate changes in affective and motivated behavior, and may be particularly involved with those that manifest differently between males and females.

Processing of the PACAP precursor by different prohormone processing enzymes leads to different mature, bioactive PACAP peptide isoforms. Processing by SPC3 (also called PC1, PC3, and BPD) leads to the PACAP‐38 isoform, whereas processing by SPC2 (also called PC2 and RPC2) leads to the PACAP‐27 isoform.^16^ While the biologically active region of PACAP lies within the PACAP‐27 sequence,^17^ there are significant differences in the distribution and stability of the two isoforms. About 90% of the total PACAP in the brain is PACAP‐38,^18^ and PACAP‐38 has been identified in numerous regions across the brain, while PACAP‐27 has only been identified in the supraoptic nucleus and paraventricular nucleus of the hypothalamus (PVN)^19^, ^20^ as well as the paraventricular nucleus of the thalamus.^14^ In regard to degradation, PACAP‐38 in human plasma has a half‐life of less than 5 min, whereas PACAP‐27 has a half‐life of over 45 min.^21^ These differences in the PACAP isoforms suggest that PACAP‐38 likely has broader but shorter‐lived effects than PACAP‐27.

## 3 EXPRESSION AND DISTRIBUTION OF THE PAC1 RECEPTOR

The PACAP isoforms bind with high affinity to three different receptors. All three receptors belong to the G‐protein‐coupled receptor superfamily with seven transmembrane domains, and all are coupled to adenylate cyclase activation.^22^ The PACAP Type I receptor, PAC1, binds PACAP with more than 100‐fold greater affinity than it does vasoactive intestinal peptide (VIP),^1^ making the PAC1 receptor selective for the PACAP isoforms. In contrast the PACAP Type II receptors, which include both the VIP 1 receptor (VPAC1) and VIP 2 receptor (VPAC2), display comparable affinity for PACAP and VIP.^1^ Because the PACAP Type I receptor is more widely expressed in the brain than the PACAP Type II receptors, which are abundant in peripheral organs,^3^ this review focuses on the PACAP Type I receptor in the brain.

As with PACAP, the PAC1 receptor in rodents has been identified in multiple limbic brain regions. While the receptor is primarily located on cell bodies and dendrites in neurons,^3^ it has also been identified on astrocytes^23^ and endothelial cells.^24^ Notably, with in situ hybridization, nearly all PACAP^+^ neurons have been found to co‐express the PAC1R,^4^ although the receptor is also found in neighboring cells and, notably, brain regions than extend beyond those with PACAP^+^ cell bodies. The brain regions where PAC1 receptor gene expression has been identified include the hypothalamus (e.g., paraventricular nucleus, supraoptic nucleus, arcuate nucleus, dorsomedial nucleus, ventromedial nucleus), thalamus (e.g., paraventricular nucleus, mediodorsal nucleus), amygdala (e.g., central nucleus), bed nucleus of the stria terminalis, nucleus accumbens, hippocampus, and locus coerulus.^4^, ^25^, ^26^ Thus, PACAP/PAC1 signaling likely occurs through longer‐distance classical neurotransmission as well as through autocrine and paracrine signaling.

Sex differences have been detected in the expression of the PAC1 receptor in the brain, likely due to the regulation of PAC1 expression. Levels of the PAC1 receptor gene in the rat have been found to be higher in females compared to males in the prefrontal cortex and hippocampus^27^, ^28^ but lower in the hypothalamus.^11^ A known regulator of PAC1 receptor transcription is ß‐estradiol. The PAC1 contains an estrogen response element upstream of the transcriptional start site,^29^, ^30^ and higher levels of serum estradiol have been found to correlate with higher levels of *PAC1* gene expression in the human, rat, and mouse brain.^29^, ^31^ Further supporting the ability of estrogen to regulate PAC1 receptor transcription, a specific allele of the PAC1 receptor, rs2267735, has been found to have less binding efficiency to the estrogen response element,^29^ and individuals expressing this allele have lower brain *PAC1* gene expression.^31^ This hormone‐dependent regulation of PAC1 receptor transcription may account for many of the sex‐related differences observed in the PACAP system.

## 4 CHARACTERISTICS OF PAC1 RECEPTOR VARIANTS

First cloned in 1993,^32^, ^33^, ^34^, ^35^ the PAC1 gene contains multiple exons that can undergo alternative splicing to generate several splice variants, the number of which differs by species. Variations can occur in the extracellular N‐terminus domain, the third intracellular loop, the transmembrane domains, and the 5′ untranslated region.^36^ In the rodent, major splice variants largely arise from variations the extracellular N‐terminus domain, through the deletion of any of several exons, and from variations in the third intracellular loop, through the addition of one of several 27‐ or 28‐amino acid cassettes.^3^ The major PAC1 variant, “null” (also sometimes called “short”), contains no insertions, is the most abundant variant in the brain, and is often used as the baseline for comparison.^36^ In the mouse, the other major variant in the brain is “hop1” (also called “hop”), which results from the addition of the 28 amino acid hop cassette into the third intracellular loop.^3^ In addition to null and hop1, the rat brain can also express the major variants “hip” from the 28 amino acid hip cassette, “hop2” from the 27 amino acid hop2 cassette, or “hiphop” from the addition of both the hip and hop1 or hip and hop2 cassettes.^3^, ^36^ The variants “short” and “very short,” resulting respectively from the deletion of exons 5–6 and exons 4–6 in the extracellular N‐terminus domain, have also been identified in rodents.^36^ Receptor variants have been identified in some of the same brain regions and even within the same cells as each other. For example, both hip and hop variants have been identified in the rat suprachiasmatic nucleus,^37^ and both null and hop have been identified within the same pyramidal cells of the rat hippocampus.^38^ It appears that the splice variants could serve somewhat distinct, rather than redundant, functions in the brain.

Although the regulation of PAC1 splicing is not well understood, a few splicing factors have been suggested. For example, the transcription factor Orthopedia (Otp) is known to target the promoter of the Ataxin 2‐Binding Protein‐1 (A2BP1/Rbfox‐1) gene, and there is an Rbfox‐1 recognition element located downstream of the hop1 encoding exon.^36^ Thus, Otp is a likely regulator of PAC1 splicing. Notably, while Otp is known to be involved in neuronal development, it has also been found to be retained in select areas of the mature mouse brain,^39^ and to be recruited in response to various stressors.^40^ In addition, calcium signaling can mediate splicing, via L‐type calcium channels and Ca^2+^/calmodulin‐dependent protein kinases IV (CaMK IV).^36^ Thus, neuronal depolarization is also likely to regulate PAC1 splicing.

Different splice variants affect different receptor dynamics, although this appears to vary across models and tissues. Variations in the extracellular N‐terminus domain largely alter ligand binding and affinity, variations in the third intracellular loop largely affect G‐protein coupling and/or interaction with other intracellular signaling proteins, variations in the transmembrane domains affect intracellular transport and heteromerization, and variations in the 5′ untranslated region affect gene expression.^36^ Primarily through in vitro testing with cell lines such as NG108‐15 (mouse neuroblastoma × rat glioma), PC‐12 (rat pheochromocytoma), and chromaffin (bovine adrenal medulla), the null variant has been found to regulate cAMP production by coupling to adenylate cyclase through Gs, and to regulate inositol phosphate (IP) production by coupling to phospholipase Cβ through Gq.^6^, ^36^ Like the null variant, the hop variant also couples to Gs and Gq subunits but additionally mobilizes Ca^2+^ through voltage‐gated Ca^2+^ channels and 2‐aminoethoxydiphenyl borate‐sensitive channels, to induce both a rapid and transient increase in Ca^2+^ through intracellular stores, and also a prolonged accumulation of Ca^2+^ through extracellular sources.^6^, ^36^ The mechanism through which these voltage‐gated Ca^2+^ channels and 2‐aminoethoxydiphenyl borate‐sensitive channels are activated has not yet been described. There is also evidence that null and hop may activate phospholipase D through Gq.^36^ In contrast, the hip cassette impairs cAMP stimulation and abolishes phospholipase C stimulation.^38^ It should be noted that combinations of alternative splicing in the extracellular N‐terminus and third intracellular loop can also occur and, in this case, variants of the extracellular N‐terminus domain can also affect intracellular signaling.^41^

Binding of the PAC1 variants also results in different intracellular signaling depending on the PACAP peptide isoform that binds to them. For both null and hop variants, binding affinity of PACAP‐38 and PACAP‐27 is roughly equal, but PACAP‐27 results in somewhat greater adenylate cyclase activation (Gs) and cAMP production, while PACAP‐38 results in much greater phospholipase C stimulation (Gq) and inositol 1,4,5‐trisphosphate (IP3) turnover and Ca^2+^ mobilization from intracellular and extracellular sources.^36^ On the other hand, the hip isoform shows lower binding affinity than null and hop for PACAP‐38 and PACAP‐27, albeit again with equal affinity for both isoforms, and PACAP‐27 results in greater adenylate cyclase activation (Gs) and cAMP production than PACAP‐38, and neither isoform activates phospholipase C stimulation (Gq) or Ca^2+^ mobilization.^36^ The hip‐hop isoform also shows similar binding affinity for PACAP‐38 and PACAP‐27, and PACAP‐27 results in greater adenylate cyclase activation (Gs) and cAMP production, PACAP‐38 results in much greater phospholipase C stimulation (Gq) and IP3 turnover, and neither isoform results in Ca^2+^ mobilization.^36^ See Table 1 for a concise description of the major spice variants in the brain and their signaling pathways.

**TABLE 1:** Major pituitary adenylate cyclase‐activating polypeptide (PACAP) type I receptor (PAC1) splice variants in the brain and their signaling pathways.

PAC1 receptor variant	Insertion into IC3 loop	cAMP production (Gs)	IP3 production (Gq)	Ca^2+^ mobilization
Null	None	PACAP‐38 ≤ PACAP‐27	PACAP‐38 ≫ PACAP‐27	PACAP‐38 > PACAP‐27
Hop1	28 amino acid hop cassette	PACAP‐38 ≤ PACAP‐27	PACAP‐38 ≫ PACAP‐27	PACAP‐38 > PACAP‐27
Hop2	27 amino acid hop2 cassette	PACAP‐38 ≤ PACAP‐27	PACAP‐38 ≫ PACAP‐27	Unknown
Hip	28 amino acid hip cassette	PACAP‐38 < PACAP‐27 (low potency)	None	None
Hiphop	Hip and hop1 or hip and hop2 cassette	PACAP‐38 < PACAP‐27 (low potency)	PACAP‐38 ≫ PACAP‐27	None

*Note*: The major PAC1 receptor splice variants in the brain differentially affect receptor dynamics depending on the PACAP peptide isoform that binds to them.

Abbreviations: cAMP, cyclic adenosine monophosphate; IC3, third intracellular loop; IP3, inositol 1,4,5‐trisphosphate.

*Source*: Information adapted from Ref. [36].

## 5 THE PACAP SYSTEM AND STRESS

Stress, originally defined by Hans Selye as a response to a demand for a change in homeostasis,^42^ is a major risk factor for anxiety, depression, and substance use.^43^ These behaviors may be adaptive in the short‐term; however, their persistence after the termination of the stressor can be problematic.^44^ The PACAP system appears to have a strong, reciprocal relationship with the stress response.

Most studies on PACAP and stress have reported an increase in PACAP gene expression and peptide levels following exposure to a stressor, particularly after chronic or repeated stress. For example, in the PVN, PACAP gene expression has been found to be upregulated in rats after chronic variable stress or injection with lipopolysaccharide or interleukin‐1 beta,^45^ although not after restraint stress or injection with hypertonic saline.^46^ Similarly, both PACAP gene expression and PACAP‐38 levels have been found to be increased in the bed nucleus of the stria terminalis of rats after chronic variable stress but not after a single day of stress,^47^, ^48^, ^49^ and PACAP fiber density in the central nucleus of the amygdala of mice is increased after partial sciatic nerve chronic constriction.^50^ Thus, PACAP appears to be upregulated in brain regions associated with the stress response.

The relationship between PACAP and stress extends to elements of the hypothalamic–pituitary–adrenal (HPA) axis. Classically, the stress response involves activation of the HPA axis, beginning with an increase in levels of corticotropin‐releasing factor (CRF) in cells of the PVN, and ending with the release of cortisol or corticosterone into the circulation. In addition to a high degree of colocalization having been found between PACAP and CRF in the rat PVN,^46^ injection of PACAP‐38 into the cerebral ventricles has been found to increase CRF gene expression and peptide levels in the rat PVN.^51^ Similarly, injection of PACAP‐38 into the rat bed nucleus of the stria terminalis or central nucleus of the amygdala, two key regions of the extended amygdala, which innervate the PVN, results in elevated circulating levels of corticosterone.^48^, ^52^ These effects on corticosterone have not been found after injection of PACAP‐38 into the rat basolateral amygdala or lateral ventricles.^48^, ^52^ Conversely, global knockout of PACAP prevents stress‐induced elevations of corticosterone. Indeed, PACAP knockout mice demonstrate diminished elevations of corticosterone after acute or repeated restraint stress and after repeated social defeat,^53^, ^54^ although not after injection with lipopolysaccharide.^53^ Together, these findings suggest that PACAP can both mediate and itself promote activation of the HPA axis.

Compared to the relationship between PACAP and stress, the relationship between the PAC1 receptor and stress is more nuanced. The PAC1 has been identified on CRF cells in the rat PVN,^55^ suggesting that it is positioned to stimulate CRF^+^ cells that control the stress response. Supporting an ability of stressor exposure to promote activity of the PAC1 system, increases in PAC1 gene expression have been observed in the rat PVN after chronic variable stress,^45^ as well as in the mouse bed nucleus of the stria terminalis, central nucleus of the amygdala, and prefrontal cortex after fear conditioning using foot shocks.^29^, ^45^ On the other hand, the number of PAC1^+^ cells has been found to be reduced in the rat central nucleus of the amygdala after acute or repeated restraint stress^56^ and gene expression of PAC1 in astrocytes of the mouse prelimbic cortex is also reduced after repeated restraint stress.^23^ These results indicate that PAC1 may be upregulated by stress exposure, but that this likely occurs in cells already expressing the PAC1 and, further, that there may be some level of negative feedback regulation between stressor exposure and the PAC1 receptor.

The specific relationship of the PAC1 with stress may be determined by the involvement of specific PAC1 splice variants. Notably, in the mouse PVN, while both the null and hop PAC1 variants show increased gene expression shortly after a combined foot shock plus restraint stress (60 min), only the hop variant demonstrates a sustained elevation after this stress (240 min).^40^ Conversely, while overexpression of the PAC1 null variant in the zebrafish preoptic area (equivalent of the mammalian PVN) results in an increase in local CRF levels, overexpression of the hop variant instead prevents a stress‐induced increase in CRF.^40^ Similarly, blockade of hop function in zebrafish larva with injection of a hop antisense morpholino leads to an increase in whole larva cortisol content,^40^ further supporting the idea that the hop variant may suppress or counteract activation of the HPA axis. While these findings have not been widely followed up, they nevertheless suggest that the PAC1 null variant could serve to promote HPA activation after exposure to a stressor, but that the hop variant may instead serve as a negative feedback signal to terminate the stress response.

## 6 THE PACAP SYSTEM AND ANXIETY

With stress being a known promoter of anxiety, a number of studies have also tied the PACAP system to anxiety‐like behavior, both following exposure to a stressor and on its own. For example, injection of PACAP‐38 into the rat bed nucleus of the stria terminalis has been found to increase startle amplitude following chronic variable stress,^57^ while sensitization of the acoustic startle response after footshock stress is inhibited by injection of a PACAP‐38 receptor antagonist in the rat bed nucleus of the stria terminalis or central nucleus of the amygdala.^58^ In addition, global knockout of PACAP in mice results in a smaller reduction in time in the light chamber of a light–dark box, time in the open sections of an elevated zero maze, and time in the open arms of an elevated plus maze following chronic social defeat.^53^ This indicates that endogenous PACAP, which shows elevated levels after stress, also promotes stress‐induced anxiety‐like behavior.

A range of anxiety‐like behaviors is also directly affected by central injection of PACAP or a PACAP receptor antagonist, chemogenetic manipulation of PACAP^+^ cells, and knockout of the PACAP gene. Rats show reduced time in the open arms of an elevated plus maze after injection of PACAP‐38 into the cerebral ventricles, bed nucleus of the stria terminalis, or central nucleus of the amygdala,^49^, ^51^, ^52^ and mice show this reduction with chemogenetic excitation of PACAP^+^ projections from the lateral parabrachial nucleus to the bed nucleus of the stria terminalis.^59^ Similarly, rats show an increase in their acoustic startle response after injection of PACAP‐38 into the bed nucleus of the stria terminalis or central nucleus of the amygdala,^58^ a reduction in time spent in the light chamber of a light–dark box after injection of PACAP‐38 into the rostral nucleus accumbens shell,^60^ and an increase in grooming and reduction in social interaction after injection of PACAP‐38 into the cerebral ventricles or PVN.^58^, ^61^, ^62^, ^63^ Conversely, PACAP knockout mice spend more time than wild‐type littermate controls in the open arms of an elevated plus maze,^64^, ^65^ spend less time in the dark chamber of a light–dark box,^66^ spend more time with a novel mouse compared to an empty cage or a familiar mouse,^65^ and spend more time in the center of an open field,^65^ and this behavior in an open field is recapitulated by injection of a PACAP‐38 receptor antagonist in the rat central nucleus of the amygdala.^50^ Thus, endogenous PACAP also promotes a range of anxiety‐like behaviors, particularly via the PVN and regions of the extended amygdala.

The limited studies in animals that have examined the role of the PAC1 receptor in anxiety‐like behavior also support a role for the endogenous PAC1 receptor system in promoting this behavior. Specifically, compared to wildtype littermate controls, mice globally lacking the PAC1 receptor spend more time in the open arms of an elevated plus maze, the open sections of an elevated zero maze, and the center of an open field.^67^ Similarly, after injection of an antisense morpholino specific for the PAC1 hop variant, zebrafish larvae show reduced anxiety‐like behavior as measured by increased time in the dark side of a light–dark arena.^40^ Together, these studies support the idea that the PAC1 receptor, and the PAC1 hop variant, acts to promote anxiety‐like behavior.

In humans, there appears to be a particular relationship between anxiety behavior and a specific allele of the PAC1 receptor gene, rs2267735. Within the putative estrogen response element of the PAC1 gene, the single‐nucleotide polymorphism that leads to the C allele has been found in a number of studies to be associated with post‐traumatic stress disorder (PTSD), which was previously classified by the Diagnostic and Statistical Manual of Mental Disorders as an Anxiety Disorder, and is now classified as a Trauma‐ and Stressor‐Related Disorder.^68^ The rs2267735 CC genotype, compared to the G allele, has reduced binding to estrogen/estrogen receptor alpha, and female carriers of the C allele show reduced gene expression of the PAC1 receptor in the brain, particularly when they have lower serum levels of estrogen.^29^, ^31^ Of relevance here, these female CC variant carriers show more symptoms of PTSD and are more likely to receive a diagnosis of PTSD when they have experienced higher levels of trauma.^31^, ^69^, ^70^, ^71^ These results do not appear to hold within the general population.^69^, ^70^, ^72^ While female carriers of the CC variant can have more PTSD symptoms and lower levels of the PAC1 in the brain, they also have higher levels of PACAP‐38 in the blood, and their cortical levels of PACAP and PAC1 gene expression are inversely correlated.^31^ Collectively, this suggests that women with PTSD exhibit dysregulation in the PACAP system, and they undergo some degree of negative feedback regulation between PACAP and the PAC1 receptor.

## 7 THE PACAP SYSTEM AND DEPRESSION

Stress is also a known promoter of depression, and studies have also connected the PACAP system with depression‐like behavior. In rodents, depression‐like behaviors are directly affected by central injection of PACAP, viral manipulation of PACAP expression, and knockout of the PACAP gene. With injection of PACAP‐38 into the lateral ventricles, male rats have been found to show increased time spent floating in a forced swim test,^55^ elevated brain reward thresholds in tests of intracranial self‐stimulation,^51^, ^58^, ^63^ and reduced intake of and preference for saccharin.^73^ These effects of PACAP on behavior in the forced swim test may occur via any of several limbic brain regions, as increased floating is similarly induced by injection of PACAP‐38 directly into the PVN of male rats,^55^ and the related behavior of reduced swimming is induced by injection of PACAP‐38 into the infralimbic cortex of male rats^74^ and by overexpression of PACAP in the paraventricular nucleus of the thalamus of female rats.^75^ Conversely, PACAP knockout mice generally show reduced depression‐like behavior, with less time spent immobile in the forced swim test,^65^ as well as less of an increase in time spent immobile after chronic social defeat.^53^

The association of the PACAP system with depression in humans is more equivocal than with anxiety. Although humans with depression symptoms do not exhibit elevated levels of PACAP‐38 in the blood,^31^ those diagnosed with major depressive disorder have been found to have elevated gene or peptide levels of PACAP in the bed nucleus of the stria terminalis, dorsolateral prefrontal cortex, and anterior cingulate cortex.^76^, ^77^ Related to the PAC1 receptor, female carriers of the risk allele, the C allele of rs2267735, also sometimes but not always have been found to show more symptoms of major depression.^31^, ^69^, ^70^, ^78^ Collectively, these findings point to a similar, albeit more circumscribed, relationship between the PACAP system and depression as between the PACAP system and anxiety.

## 8 SIMILARITIES IN THE PACAP SYSTEM ACROSS STRESS, ANXIETY, AND DEPRESSION

The literature on stress, anxiety, and depression collectively aligns to identify several general themes for the PACAP system in relation to affective behavior. Noting that stress is a major risk factor for anxiety and depression,^43^ exposure to a stressor itself is generally found to increase levels of both PACAP and PAC1, particularly after chronic or repeated stressor exposure. These upregulated levels of PACAP and PAC1 can be found not only in the PVN, the origin of HPA axis activation, but also in regions of the extended amygdala (e.g., the bed nucleus of the stria terminalis, central nucleus of the amygdala, and nucleus accumbens shell), which innervate the PVN.^29^, ^45^, ^47^, ^48^, ^49^, ^50^ The increase in PACAP and PAC1 levels can also be found in limbic brain regions that connect with the extended amygdala, such as the prefrontal cortex.^29^ Stressor exposure can also upregulate multiple variants of the PAC1 receptor, and this appears to be time‐dependent, with the hop variant demonstrating a more sustained upregulation than the null variant after stressor exposure.^40^ Examining behavioral effects of these changes after stressor exposure, elevations in PACAP and PAC1 levels can promote both anxiety‐like behavior and depression‐like behavior and, as with stressor exposure, this can occur via the PVN, regions of the extended amygdala (bed nucleus of the stria terminalis, central nucleus of the amygdala, and nucleus accumbens shell), and connected limbic regions such as prefrontal cortex and paraventricular nucleus of the thalamus.^55^, ^58^, ^60^, ^74^, ^75^ The PAC1 hop variant may reduce rather than promote anxiety‐like behavior.^40^ Stress, anxiety, and depression exhibit a complex relationship with the motivated behavior of drug use, being found to inhibit or promote drug use depending on the context.^79^, ^80^ As such, the PACAP system might also be expected to exhibit a dynamic relationship with the use of drugs such as alcohol.

## 9 THE PACAP SYSTEM AND ALCOHOL

Alcohol use disorder (AUD) is characterized by an impaired ability to stop or control the use of alcohol despite adverse consequences.^81^ While individuals can have an AUD without meeting the criteria for alcohol dependence, binge drinking is one vector through which alcohol dependence can be established.^81^ In rodents, a commonly employed model of binge drinking is the intermittent access two‐bottle‐choice procedure.^82^, ^83^ Under this procedure, rats or mice are offered 10%–20% ethanol, typically for 24‐h periods, 3 days per week (e.g., Monday, Wednesday, and Friday), with water and chow available ad libitum.^82^, ^83^, ^84^ Drinking under this model leads to blood ethanol levels that are considered pharmacologically relevant (typically 50–100 mg/dL), but are not typically sufficient to induce ethanol dependence.^82^, ^85^ One common means to induce ethanol dependence is chronic intermittent ethanol (CIE) exposure to ethanol vapor (often 14 h on/10 h off), with target blood ethanol levels in the range of intoxication (typically 150–250 mg/dL).^86^ According to the “dark side of addiction” hypothesis, drug consumption is often initiated due to the hedonic experiences of drug use (positive reinforcement) but, with the development of dependence, is continued in order to avoid the aversive experiences of withdrawal (negative reinforcement).^87^ In this regard, the intermittent access procedure can be viewed as a model of early to transitional stages of ethanol drinking, and the CIE vapor exposure procedure can be viewed as inducing dependence‐level ethanol drinking.

A major neurobiological driver of the aversive experiences of withdrawal from ethanol is believed to be neuropeptide systems that are involved in stress.^88^ In general, both PACAP and PAC1 expression are increased in the PVN and extended amygdala following exposure to a stressor (e.g.,^29^, ^45^, ^47^, ^49^, ^89^) and promote activation of the HPA axis (e.g.,^48^, ^51^, ^52^), and the PAC1 hop variant demonstrates an extended increase in expression and may serve as a negative feedback signal to terminate the stress response.^40^ Further, PACAP in the PVN and extended amygdala also promotes anxiety‐like behavior (e.g.,^49^, ^51^, ^52^) likely via the PAC1 receptor^67^ and possibly specifically via the PAC1 hop variant,^40^ and PACAP in the PVN and other limbic areas promotes depression‐like behavior (e.g.,^55^, ^73^, ^75^), possibly also via the PAC1 receptor.^78^ Collectively, this supports the idea that the function of the PACAP system could be promoted by the aversive experience of withdrawal from alcohol and in turn contribute to behavior that results from that experience.

Studies on PACAP and alcohol have reported an increase in PACAP gene expression and peptide levels following ethanol exposure and also following withdrawal from ethanol, although these increases occur only in select brain regions. After 4 weeks of drinking under the intermittent access procedure, and after the start of their daily ethanol access, male rats show increased PACAP gene expression in the paraventricular nucleus of the thalamus but not the PVN.^90^ They also show increased levels of PACAP‐27 in the paraventricular nucleus of the thalamus, despite no increase in the number of cells expressing this peptide.^90^ Similarly, during acute withdrawal from ethanol after CIE exposure or 7 weeks of drinking under the intermittent access procedure, male rats and male and female mice show increased levels of PACAP‐38 in the bed nucleus of the stria terminalis but not the central nucleus of the amygdala.^15^, ^91^ Together, these findings suggest that PACAP is upregulated in select limbic brain regions following ethanol intake and ethanol withdrawal.

Similar to the relationship between PACAP and ethanol, studies have reported an increase in PAC1 receptor expression following ethanol exposure. Specifically, PAC1 gene expression has been found to be increased in C6 glioma cells following acute exposure to ethanol,^92^ and PAC1 cell labeling has been found to be increased in the nucleus accumbens core but not shell of male rats with a history of higher‐ compared to lower‐level operant ethanol self‐administration.^93^ Notably, alcohol consumption can lead to an increase in estradiol levels in both women and men,^94^, ^95^ so this could be a mechanism through which this upregulation occurs. On the other hand, the number of PAC1^+^ cells has not been found to be increased in the bed nucleus of the stria terminalis of male rats or male and female mice during acute ethanol withdrawal after CIE exposure or after 7 weeks of drinking under the intermittent access procedure.^15^, ^91^ Given that several prior studies have reported no change in the number of PACAP^+^ or PAC1^+^ cells despite a change in gene expression or peptide levels, it is unclear if this lack of change in PAC1^+^ cell number after ethanol withdrawal occurred despite a change in PAC1 expression or if it indicates a more general lack of change in the PAC1 system during ethanol withdrawal.

Several studies using the intermittent access procedure or models of lower‐level ethanol drinking have found that PACAP can reduce or inhibit ethanol drinking. For example, overexpression of PACAP in the paraventricular nucleus of the thalamus of female rats has been found to prevent the normal escalation of ethanol drinking across weeks under the intermittent access procedure, with no change in sucrose drinking.^75^ Similarly, injection of PACAP‐27 in the rostral nucleus accumbens shell of male and female rats has been found to reduce binge‐like drinking under the intermittent access procedure, again with no change in sucrose drinking,^60^ and injection of PACAP‐38 in the nucleus accumbens core of male rats has also been found to reduce drinking under the intermittent access procedure.^60^ Conversely, injection of a PACAP‐27 receptor antagonist into the rostral nucleus accumbens shell of male rats has been found to stimulate drinking under the intermittent access procedure^60^ and global knockout of PACAP in male mice has been found to result in higher levels of drinking of ethanol but not sucrose when provided with ad libitum access.^96^

The effect of PACAP on ethanol drinking appears to change after higher levels of ethanol exposure so that, in line with the dark side of addiction hypothesis, PACAP comes to promote rather than suppress excessive ethanol drinking. For example, in selectively‐bred Sardinian alcohol‐preferring but not outbred Wistar male rats, injection of a PACAP‐38 receptor antagonist into the cerebral ventricles or nucleus accumbens core but not shell reduces excessive operant ethanol self‐administration.^93^ This same injection into the cerebral ventricles has no effect on saccharin or sucrose self‐administration.^93^ Similarly, injection of a PACAP‐38 receptor antagonist into the bed nucleus of the stria terminalis reduces excessive operant ethanol self‐administration of outbred Wistar male rats after CIE exposure but has no effect in non‐dependent control rats.^91^ Notably, this same injection also reverses the ethanol withdrawal‐induced reduction in time spent in the light compartment of a light–dark box after CIE exposure.^91^ Under the intermittent access procedure, chemogenetic inhibition of PACAP^+^ inputs into the bed nucleus of the stria terminalis of male and female mice also suppresses ethanol drinking^15^ while injection of PACAP‐27 into the caudal nucleus accumbens shell of male rats conversely stimulates it.^60^ All together, while these results suggest that PACAP may have site‐specific effects on ethanol drinking, they also suggest that PACAP fits within the dark side of addiction framework, and could act as a stress‐related neuropeptide to ultimately drive ethanol intake through negative reinforcement.

Limited evidence suggests that the role of the PAC1 receptor in ethanol drinking may depend more on brain region than on level of ethanol exposure, although this could also reflect regional differences in expression of PAC1 receptor variants. In male Sardinian alcohol‐preferring rats, short‐hairpin RNA knockdown of the PAC1 in the nucleus accumbens core reduces excessive operant ethanol self‐administration,^93^ similar to effects of injection of a PACAP‐38 receptor antagonist into the nucleus accumbens core.^93^ In contrast, this same PAC1 knockdown in the nucleus accumbens shell of male Sardinian alcohol‐preferring rats instead promotes their ethanol self‐administration,^97^ just as injection of a PACAP‐27 receptor antagonist stimulated drinking under the intermittent access procedure.^60^

The PACAP system also appears to be associated with alcohol drinking in humans. Notably, a specific allele of PACAP, rs2856966, has been associated with social alcohol consumption within a Finnish general adult population,^98^ and a single‐nucleotide polymorphism of the PAC1 gene, rs2302475, has been associated with problematic alcohol use in young adult Polish, Ukrainian, and Swedish women (rs2267735 was not tested).^99^ These results strongly implicate the PACAP system in alcohol use and likely in alcohol use disorder.

## 10 A PROPOSED RELATIONSHIP BETWEEN THE PAC1 AND MOTIVATED BEHAVIOR

In light of evidence tying the PACAP system with stress, and the hypothesized relationship between stress and the development of drug dependence (as proposed in the “dark side of addiction” hypothesis), we propose here a potential relationship between the PACAP system and the motivated behavior of drug use. We see that both PACAP and PAC1 expression are increased in select limbic areas following exposure to a stressor^29^, ^45^, ^47^, ^49^, ^89^ and following intake and/or withdrawal from the drug ethanol.^15^, ^90^, ^91^, ^92^, ^93^ We also see in one study that the PAC1 hop variant demonstrates an extended increase in expression after exposure to a stressor, and may serve to terminate the physiological response to stress.^40^ Further, this increase in PACAP and the PAC1 receptor, possibly including the hop variant, appears to then serve to promote stress‐related behavioral responses such as anxiety‐like behavior and depression‐like behavior,^40^, ^49^, ^51^, ^52^, ^55^, ^67^, ^73^, ^75^, ^78^ which can be adaptive in the short‐term but become problematic when persistent.^44^ We see that with a lower level of ethanol exposure, PACAP can suppress ethanol drinking.^60^, ^75^, ^96^ Conversely, with a higher level of ethanol exposure, and the development of ethanol dependence, PACAP and the PAC1 receptor can instead promote further ethanol intake,^15^, ^91^, ^93^ in addition to promoting ethanol withdrawal‐induced anxiety‐like behavior.^91^ Drawing a parallel between the motivated behaviors of ethanol drinking and palatable food intake,^80^ we note that the relationship between stress and palatable food eating is known to follow an inverted U pattern, such that lower perceived levels of stress can promote the consumption of palatable foods, while higher perceived levels instead inhibit it.^79^

**FIGURE 1:**
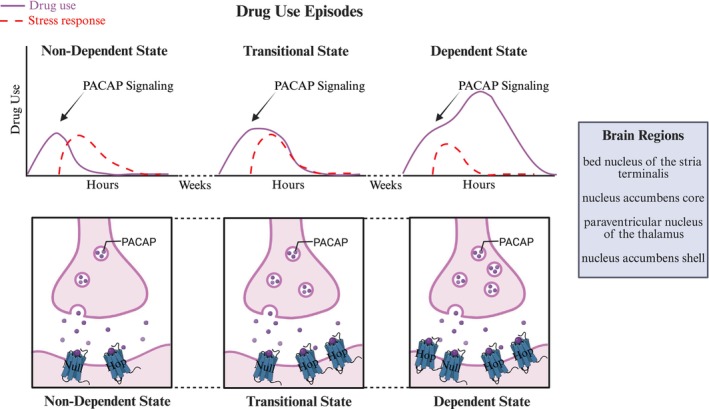
Diagram illustrating the proposed model for a relationship between the pituitary adenylate cyclase‐activating polypeptide (PACAP) system and the motivated behavior of drug use. Non‐dependent, lower‐level drug use causes the release of PACAP onto post‐synaptic neurons that contain multiple PACAP receptor variants, including both null and hop, which serve to induce a stress response and limit the drug intake. Over time, with repeated episodes of drug use and withdrawal, the hop variant receptors are upregulated in these same post‐synaptic neurons, such that drug use in a dependent state causes the release of PACAP onto neurons that contain higher levels of hop receptor variants, which serve to truncate the stress response and facilitate additional drug use within individual drug use episodes. Brain regions where this may occur include the bed nucleus of the stria terminalis, nucleus accumbens core, and paraventricular nucleus of the thalamus, and possibly also the nucleus accumbens shell. Created with BioRender.com.

In the context of these published findings, and noting that this remains speculative, we therefore propose the following model for a relationship between the PACAP system and the motivated behavior of drug use (Figure 1): Early, lower‐level drug use causes the release of PACAP onto post‐synaptic neurons that contain multiple PAC1 receptor variants, including both null and hop, which initially serves to limit the drug intake, possibly due to perception by the system as a high stress challenge. Over time, as the drug leaves the system, levels of PACAP itself are upregulated and PAC1 hop variant receptors are upregulated in those same post‐synaptic neurons, which acts to promote allostasis and counteract the effects of the drug withdrawal. As a result, during subsequent drug use, the binding of PACAP onto these hop receptors is perceived by the system as a lower stress challenge, allowing for additional drug use within individual drug use episodes. That is, the upregulation of the hop relative to the null receptor variants during repeated episodes of drug use and withdrawal serves to reverse the effects of PACAP on drug use, switching from negative feedback in a non‐dependent state to positive feedback in a dependent state. In this way, a similar neuropeptide response to the stimulus of drug use eventually results in dissimilar, and even opposite, downstream effects on post‐synaptic neurons and organism behavior.

## 11 CONCLUSIONS

The PAC1 receptor and its splice variants has been characterized in the fields of stress, anxiety, and depression, but has received far more limited attention in the field of motivated behavior. The growing literature on the role of the PACAP system in alcohol use suggests that this relationship may be similar to that of the PACAP system and stress. Specifically, it has been shown that PACAP can either inhibit or promote ethanol drinking depending on the level of ethanol exposure and/or dependent state of the animal. We speculate that this may be due to ethanol‐induced changes in the expression of specific PAC1 receptor variants in the limbic system and, in particular, in the extended amygdala and related structures. It is likely that this phenomenon can also be found with other abused drugs. In light of the paucity of studies on this topic, we propose that the PAC1 splice variants should be thoroughly examined in the context of motivated behavior, as they may prove to be effective medication targets for the treatment of drug use disorders.

## AUTHOR CONTRIBUTIONS

**Brody A. Carpenter:** Funding acquisition; writing – original draft; writing – review and editing; conceptualization. **Jessica R. Barson:** Finding acquisition; writing – original draft; writing – review and editing; conceptualization.

## CONFLICT OF INTEREST STATEMENT

The authors declare no conflicts of interest.

## ETHICS STATEMENT

This work was supported by the National Institute on Alcohol Abuse and Alcoholism under Award Numbers R01AA028218 (J.R.B.), and F31AA031427 (B.A.C). The content is solely the responsibility of the authors and does not necessarily represent the official views of the NIH.

## Data Availability

Data sharing not applicable to this article as no datasets were generated or analysed during the current study.
